# Upward closed talocrural dislocation without fracture

**DOI:** 10.11604/pamj.2017.26.179.9562

**Published:** 2017-03-29

**Authors:** Bah Aliou, Beaudouin Emmanuel

**Affiliations:** 1 Orthopedic and Trauma Surgery Department at Chambery Hospital Metropole Savoie, France; 2 Hospital Practitioner in Orthopedic and Trauma Department at Chambery Hospital Metropole Savoie, France

**Keywords:** Ankle dislocation, syndesmosis trauma, talocrural dislocation

## Image in medicine

The ankle dislocations are very rare, around 1% of all dislocations. It is usually a fracture-dislocation. The talocrural dislocations are exceptional. We report a case about a 63 years male, carpenter, who fell off the stairs leading to an axial compression trauma of his left ankle. In clinical examination, we found a deformation of his ankle without sensory or vascular deficit. This trauma was closed. The x-rays (A, with arrow) found upward talocrural dislocation without fracture. Immediate reduction was performed. The articulation was still incoercible and unstable. We set up two 3.5 mm tri cortical screws in compression followed by six weeks of cast immobilization (B). After 6 weeks, an X-ray was performed where we noticed a decline off the screws (C, with arrow) without clinical impact. We proposed a removal of materiel, but he refused. After 36 months of follow up, functional results were satisfactory.

**Figure 1:**
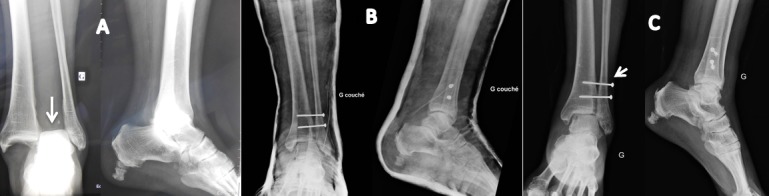
(A) ankle X-ray showed the upward talocrural dislocation (white arrow); (B) X-ray after immediate reduction with two screws and immobilization with cast; (C) ankle X-ray showed the decline off screws (white Arrow)

